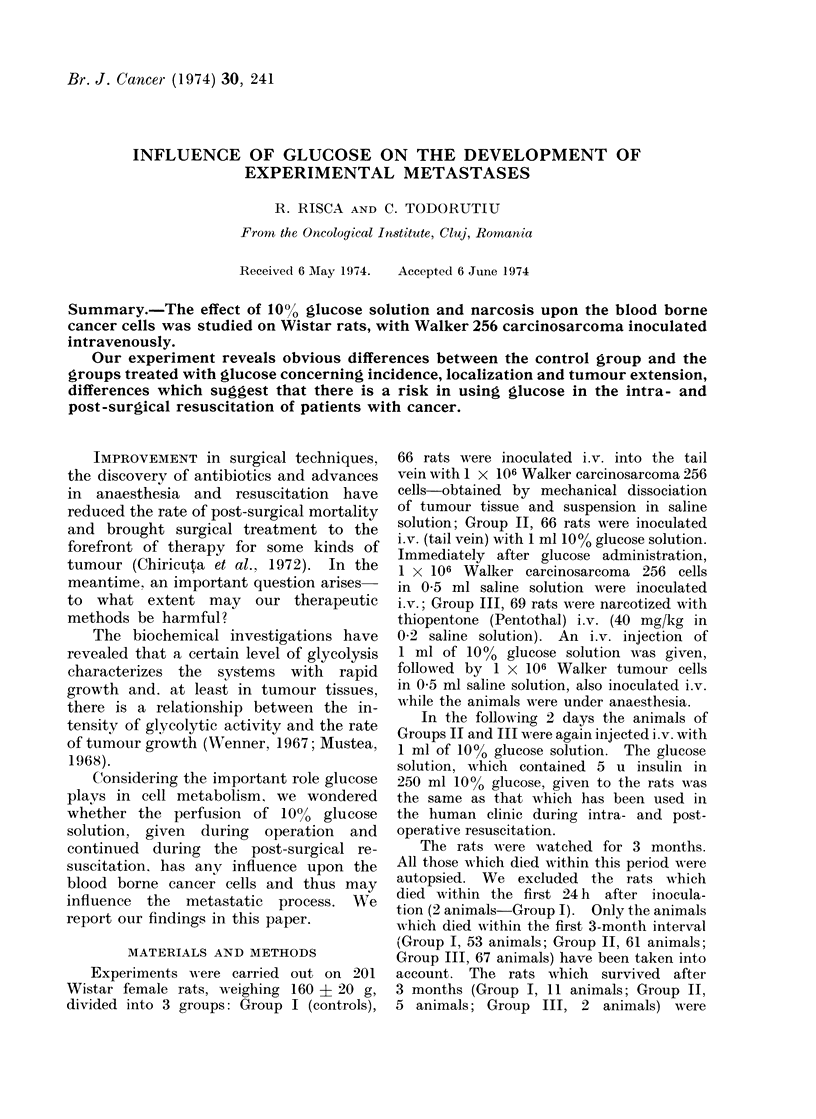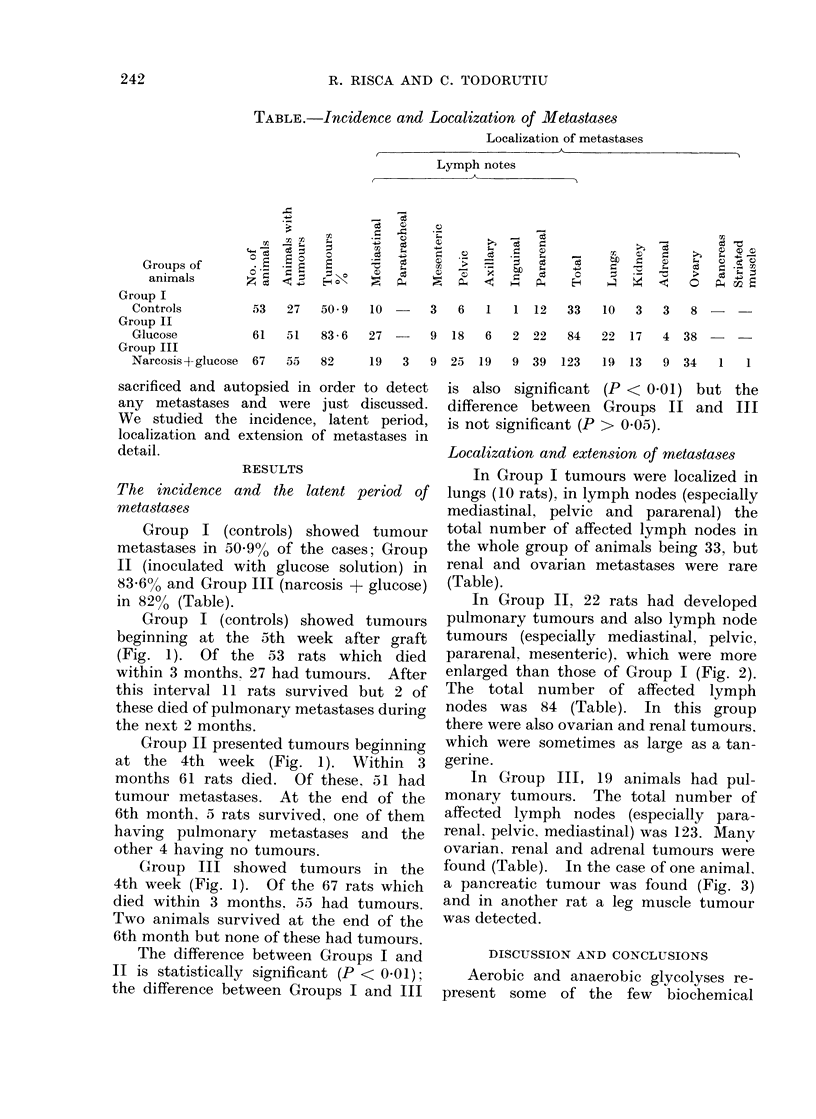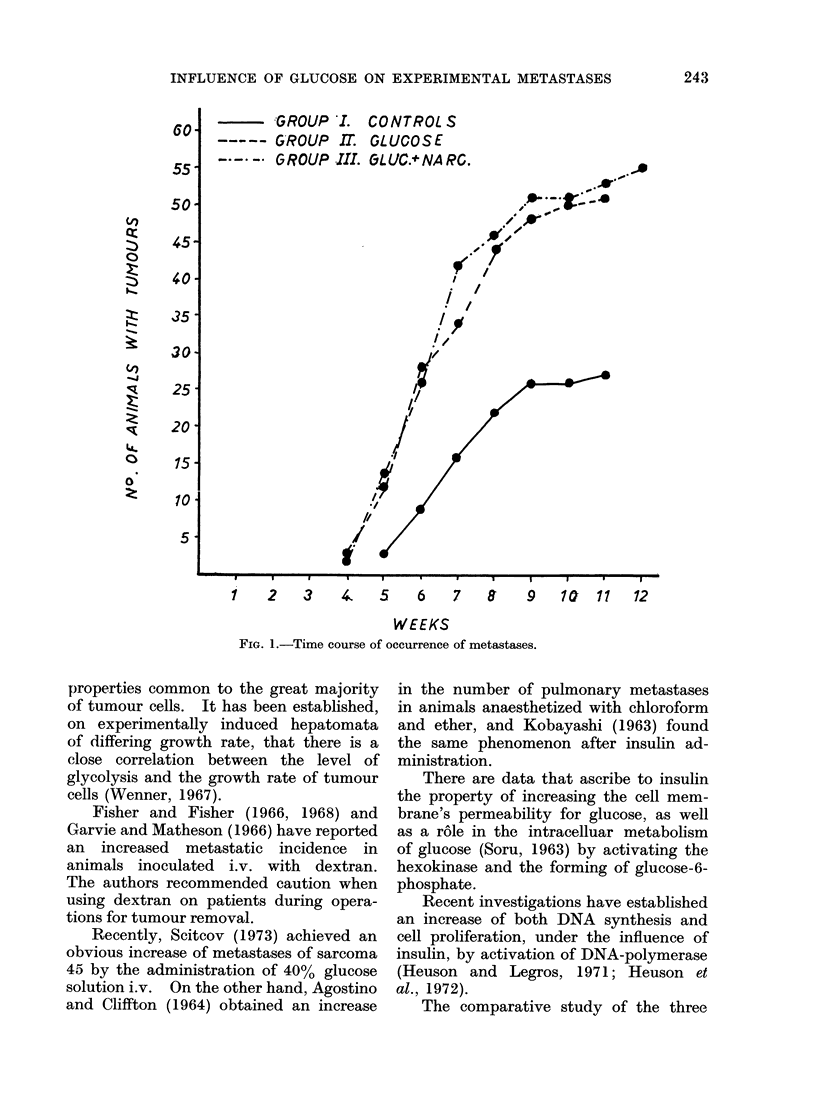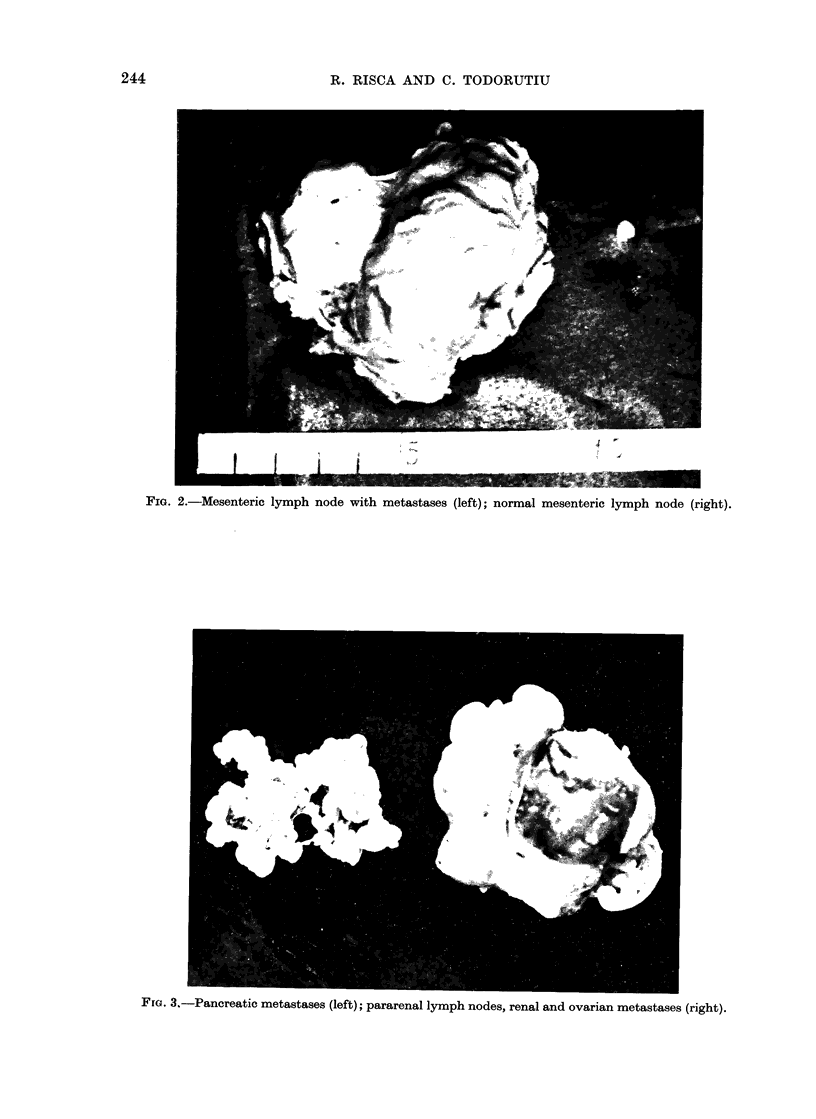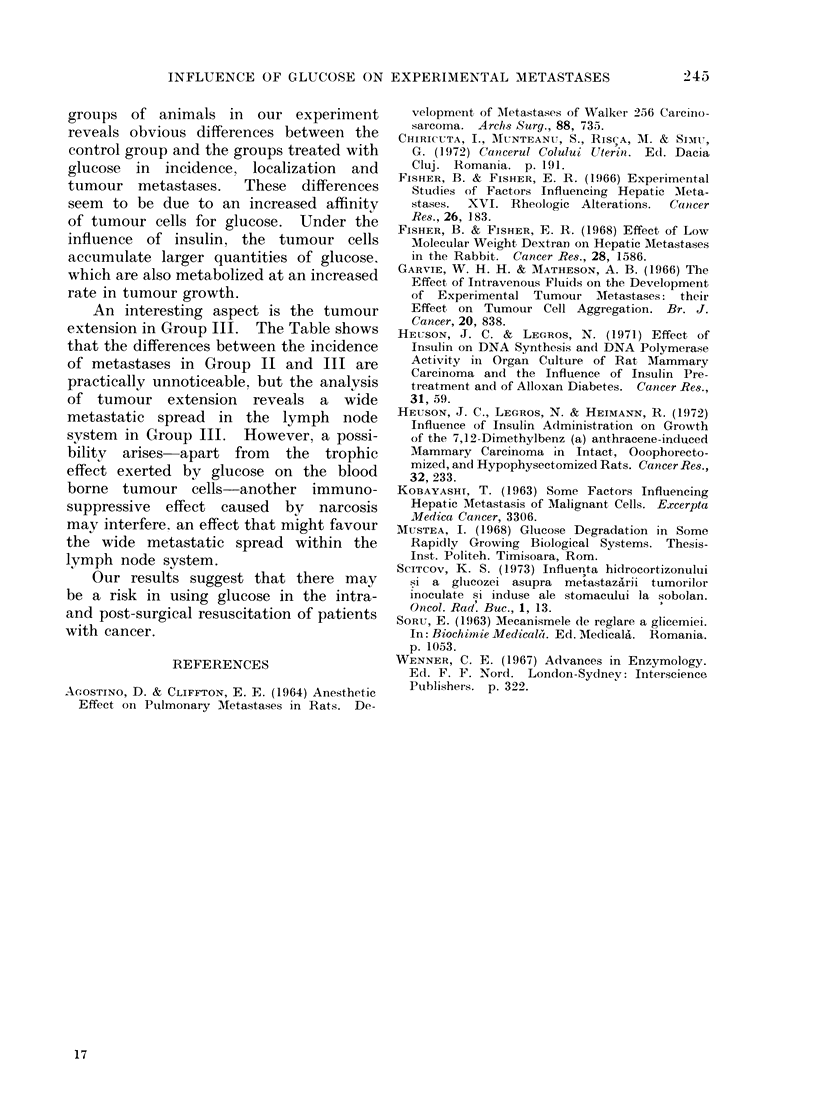# Influence of Glucose on the Development of Experimental Metastases

**DOI:** 10.1038/bjc.1974.188

**Published:** 1974-09

**Authors:** R. Risca, C. Todorutiu

## Abstract

**Images:**


*Br. J. Cancer* (1974) **30**, 241

### INFLUENCE OF GLUCOSE ON THE DEVELOPMENT OF EXPERIMENTAL METASTASES

R. RISCA AND C. TODORUTIU

*From the Oncological Institute, Cluj, Romania*

Received 6 May 1974. Accepted 6 June 1974

**Summary.—The effect of 10% glucose solution and narcosis upon the blood borne cancer cells was studied on Wistar rats, with Walker 256 carcinosarcoma inoculated intravenously.**

**Our experiment reveals obvious differences between the control group and the groups treated with glucose concerning incidence, localization and tumour extension, differences which suggest that there is a risk in using glucose in the intra- and post-surgical resuscitation of patients with cancer.**

IMPROVEMENT in surgical techniques, the discovery of antibiotics and advances in anaesthesia and resuscitation have reduced the rate of post-surgical mortality and brought surgical treatment to the forefront of therapy for some kinds of tumour (Chiricuţa *et al.*, 1972). In the meantime, an important question arises—to what extent may our therapeutic methods be harmful?

The biochemical investigations have revealed that a certain level of glycolysis characterizes the systems with rapid growth and, at least in tumour tissues, there is a relationship between the intensity of glycolytic activity and the rate of tumour growth (Wenner, 1967; Mustea, 1968).

Considering the important role glucose plays in cell metabolism, we wondered whether the perfusion of 10% glucose solution, given during operation and continued during the post-surgical resuscitation, has any influence upon the blood borne cancer cells and thus may influence the metastatic process. We report our findings in this paper.

### MATERIALS AND METHODS

Experiments were carried out on 201 Wistar female rats, weighing $160 \pm 20$ g, divided into 3 groups: Group I (controls), 66 rats were inoculated i.v. into the tail vein with $1 \times 10^6$ Walker carcinosarcoma 256 cells—obtained by mechanical dissociation of tumour tissue and suspension in saline solution; Group II, 66 rats were inoculated i.v. (tail vein) with 1 ml 10% glucose solution. Immediately after glucose administration, $1 \times 10^6$ Walker carcinosarcoma 256 cells in 0·5 ml saline solution were inoculated i.v.; Group III, 69 rats were narcotized with thiopentone (Pentothal) i.v. (40 mg/kg in 0·2 saline solution). An i.v. injection of 1 ml of 10% glucose solution was given, followed by $1 \times 10^6$ Walker tumour cells in 0·5 ml saline solution, also inoculated i.v. while the animals were under anaesthesia.

In the following 2 days the animals of Groups II and III were again injected i.v. with 1 ml of 10% glucose solution. The glucose solution, which contained 5 u insulin in 250 ml 10% glucose, given to the rats was the same as that which has been used in the human clinic during intra- and post-operative resuscitation.

The rats were watched for 3 months. All those which died within this period were autopsied. We excluded the rats which died within the first 24 h after inoculation (2 animals—Group I). Only the animals which died within the first 3-month interval (Group I, 53 animals; Group II, 61 animals; Group III, 67 animals) have been taken into account. The rats which survived after 3 months (Group I, 11 animals; Group II, 5 animals; Group III, 2 animals) were

TABLE.—*Incidence and Localization of Metastases*

| | | | | Localization of metastases | | | | | | | | | | | | | |
|---|---|---|---|---|---|---|---|---|---|---|---|---|---|---|---|---|---|
| | | | | Lymph notes | | | | | | | | | | | | | |
| Groups of animals | No. of animals | Animals with tumours | Tumours % | Mediastinal | Paratracheal | Mesenteric | Pelvic | Axillary | Inguinal | Pararenal | Total | Lungs | Kidney | Adrenal | Ovary | Pancreas | Striated muscle |
| Group I Controls | 53 | 27 | 50·9 | 10 | — | 3 | 6 | 1 | 1 | 12 | 33 | 10 | 3 | 3 | 8 | — | — |
| Group II Glucose | 61 | 51 | 83·6 | 27 | — | 9 | 18 | 6 | 2 | 22 | 84 | 22 | 17 | 4 | 38 | — | — |
| Group III Narcosis + glucose | 67 | 55 | 82 | 19 | 3 | 9 | 25 | 19 | 9 | 39 | 123 | 19 | 13 | 9 | 34 | 1 | 1 |

sacrificed and autopsied in order to detect any metastases and were just discussed. We studied the incidence, latent period, localization and extension of metastases in detail.

### RESULTS

### *The incidence and the latent period of metastases*

Group I (controls) showed tumour metastases in 50·9% of the cases; Group II (inoculated with glucose solution) in 83·6% and Group III (narcosis + glucose) in 82% (Table).

Group I (controls) showed tumours beginning at the 5th week after graft (Fig. 1). Of the 53 rats which died within 3 months, 27 had tumours. After this interval 11 rats survived but 2 of these died of pulmonary metastases during the next 2 months.

Group II presented tumours beginning at the 4th week (Fig. 1). Within 3 months 61 rats died. Of these, 51 had tumour metastases. At the end of the 6th month, 5 rats survived, one of them having pulmonary metastases and the other 4 having no tumours.

Group III showed tumours in the 4th week (Fig. 1). Of the 67 rats which died within 3 months, 55 had tumours. Two animals survived at the end of the 6th month but none of these had tumours.

The difference between Groups I and II is statistically significant ($P < 0{\cdot}01$); the difference between Groups I and III is also significant ($P < 0{\cdot}01$) but the difference between Groups II and III is not significant ($P > 0{\cdot}05$).

### *Localization and extension of metastases*

In Group I tumours were localized in lungs (10 rats), in lymph nodes (especially mediastinal, pelvic and pararenal) the total number of affected lymph nodes in the whole group of animals being 33, but renal and ovarian metastases were rare (Table).

In Group II, 22 rats had developed pulmonary tumours and also lymph node tumours (especially mediastinal, pelvic, pararenal, mesenteric), which were more enlarged than those of Group I (Fig. 2). The total number of affected lymph nodes was 84 (Table). In this group there were also ovarian and renal tumours, which were sometimes as large as a tangerine.

In Group III, 19 animals had pulmonary tumours. The total number of affected lymph nodes (especially pararenal, pelvic, mediastinal) was 123. Many ovarian, renal and adrenal tumours were found (Table). In the case of one animal, a pancreatic tumour was found (Fig. 3) and in another rat a leg muscle tumour was detected.

### DISCUSSION AND CONCLUSIONS

Aerobic and anaerobic glycolyses represent some of the few biochemical

FIG. 1.—Time course of occurrence of metastases.

properties common to the great majority of tumour cells. It has been established, on experimentally induced hepatomata of differing growth rate, that there is a close correlation between the level of glycolysis and the growth rate of tumour cells (Wenner, 1967).

Fisher and Fisher (1966, 1968) and Garvie and Matheson (1966) have reported an increased metastatic incidence in animals inoculated i.v. with dextran. The authors recommended caution when using dextran on patients during operations for tumour removal.

Recently, Scitcov (1973) achieved an obvious increase of metastases of sarcoma 45 by the administration of 40% glucose solution i.v. On the other hand, Agostino and Cliffton (1964) obtained an increase in the number of pulmonary metastases in animals anaesthetized with chloroform and ether, and Kobayashi (1963) found the same phenomenon after insulin administration.

There are data that ascribe to insulin the property of increasing the cell membrane's permeability for glucose, as well as a rôle in the intracelluar metabolism of glucose (Soru, 1963) by activating the hexokinase and the forming of glucose-6-phosphate.

Recent investigations have established an increase of both DNA synthesis and cell proliferation, under the influence of insulin, by activation of DNA-polymerase (Heuson and Legros, 1971; Heuson *et al.*, 1972).

The comparative study of the three

Fig. 2.—Mesenteric lymph node with metastases (left); normal mesenteric lymph node (right).

Fig. 3.—Pancreatic metastases (left); pararenal lymph nodes, renal and ovarian metastases (right).

groups of animals in our experiment reveals obvious differences between the control group and the groups treated with glucose in incidence, localization and tumour metastases. These differences seem to be due to an increased affinity of tumour cells for glucose. Under the influence of insulin, the tumour cells accumulate larger quantities of glucose, which are also metabolized at an increased rate in tumour growth.

An interesting aspect is the tumour extension in Group III. The Table shows that the differences between the incidence of metastases in Group II and III are practically unnoticeable, but the analysis of tumour extension reveals a wide metastatic spread in the lymph node system in Group III. However, a possibility arises—apart from the trophic effect exerted by glucose on the blood borne tumour cells—another immunosuppressive effect caused by narcosis may interfere, an effect that might favour the wide metastatic spread within the lymph node system.

Our results suggest that there may be a risk in using glucose in the intra- and post-surgical resuscitation of patients with cancer.

### REFERENCES

AGOSTINO, D. & CLIFFTON, E. E. (1964) Anesthetic Effect on Pulmonary Metastases in Rats. Development of Metastases of Walker 256 Carcinosarcoma. *Archs Surg.*, **88**, 735.

CHIRICUTA, I., MUNTEANU, S., RISÇA, M. & SIMU, G. (1972) *Cancerul Colului Uterin.* Ed. Dacia Cluj. Romania. p. 191.

FISHER, B. & FISHER, E. R. (1966) Experimental Studies of Factors Influencing Hepatic Metastases. XVI. Rheologic Alterations. *Cancer Res.*, **26**, 183.

FISHER, B. & FISHER, E. R. (1968) Effect of Low Molecular Weight Dextran on Hepatic Metastases in the Rabbit. *Cancer Res.*, **28**, 1586.

GARVIE, W. H. H. & MATHESON, A. B. (1966) The Effect of Intravenous Fluids on the Development of Experimental Tumour Metastases: their Effect on Tumour Cell Aggregation. *Br. J. Cancer*, **20**, 838.

HEUSON, J. C. & LEGROS, N. (1971) Effect of Insulin on DNA Synthesis and DNA Polymerase Activity in Organ Culture of Rat Mammary Carcinoma and the Influence of Insulin Pretreatment and of Alloxan Diabetes. *Cancer Res.*, **31**, 59.

HEUSON, J. C., LEGROS, N. & HEIMANN, R. (1972) Influence of Insulin Administration on Growth of the 7,12-Dimethylbenz (a) anthracene-induced Mammary Carcinoma in Intact, Ooophorectomized, and Hypophysectomized Rats. *Cancer Res.*, **32**, 233.

KOBAYASHI, T. (1963) Some Factors Influencing Hepatic Metastasis of Malignant Cells. *Excerpta Medica Cancer*, 3306.

MUSTEA, I. (1968) Glucose Degradation in Some Rapidly Growing Biological Systems. Thesis-Inst. Politeh. Timisoara, Rom.

SCITCOV, K. S. (1973) Influenţa hidrocortizonului şi a glucozei asupra metastazării tumorilor inoculate şi induse ale stomacului la şobolan. *Oncol. Rad. Buc.*, **1**, 13.

SORU, E. (1963) Mecanismele de reglare a glicemiei. In: *Biochimie Medicală*. Ed. Medicală. Romania. p. 1053.

WENNER, C. E. (1967) Advances in Enzymology. Ed. F. F. Nord. London-Sydney: Interscience Publishers. p. 322.